# Degradable multi-arm PEG hydrogels with tunable stiffness and diffusivity

**DOI:** 10.1039/d6bm00440g

**Published:** 2026-06-17

**Authors:** Kristie Cheng, Evelyn Lim, Brett Stern, Janet Zoldan, Nicholas Peppas

**Affiliations:** a Department of Biomedical Engineering, The University of Texas at Austin Austin TX Texas USA zjanet@utexas.edu peppas@che.utexas.edu; b McKetta Department of Chemical Engineering, The University of Texas at Austin Austin Texas USA; c Institute for Biomaterials, Drug Delivery, and Regenerative Medicine, The University of Texas at Austin Texas USA; d Department of Pediatrics, Dell Medical School Austin Texas USA; e Department of Surgery and Perioperative Care, Dell Medical School, The University of Texas at Austin Austin Texas USA; f Division of Molecular Pharmaceutics and Drug Delivery, College of Pharmacy, The University of Texas at Austin Austin Texas USA

## Abstract

The bone marrow extracellular matrix (BM-ECM) has distinct mechanical and biochemical subniches, in which hematopoietic stem cells (HSCs) reside, self-renew, and differentiate into immune cells. Stiffness and diffusivity are key mechano-regulators of HSC fate. To control these key microenvironment parameters, we prepared a poly(ethylene-glycol) norbornene (PEGNB)-modified hydrogel platform with tunable stiffness and solute diffusivity by varying the polymer volume fraction, the number of macromolecular arms, their arm length, and their crosslinker type. The latter was identified as either nondegradable dithiothreitol (DTT) or matrix metalloproteinase (MMP)-degradable peptides. We characterized 24 PEGNB hydrogels for stiffness, swelling, and solute diffusion. Hydrogel swelling ratios ranged from 0.89 to 2.48 for DTT-crosslinked networks and 1.49 to 4.86 for peptide-crosslinked networks, supporting solvent retention for cell culture. Storage moduli ranged from 6.4 to 44.8 kPa (DTT-crosslinked networks) and 3.6 to 32.7 kPa (peptide-crosslinked networks), within the physiological range of the BM-ECM. Solute diffusivity values were also evaluated for all hydrogel formulations. Introduction of an MMP-sensitive crosslinker maintained the same relationship among hydrogel stiffness, swelling and solute diffusion while allowing for cell-mediated remodeling and high cell viability. Unlike prior predictive models, our study accounts for structural complexity to better match *in vivo* conditions. Therefore, we present a modular framework for engineering PEGNB hydrogels with independently tunable mechanical and transport properties, providing a robust, physiologically relevant platform to investigate stem cell–matrix interactions and advance stem cell-based tissue engineering.

## Abstract 2

Modular PEGNB hydrogels recapitulate mechanically relevant bone marrow microenvironments for 3D stem cell culture.

## Graphical abstract

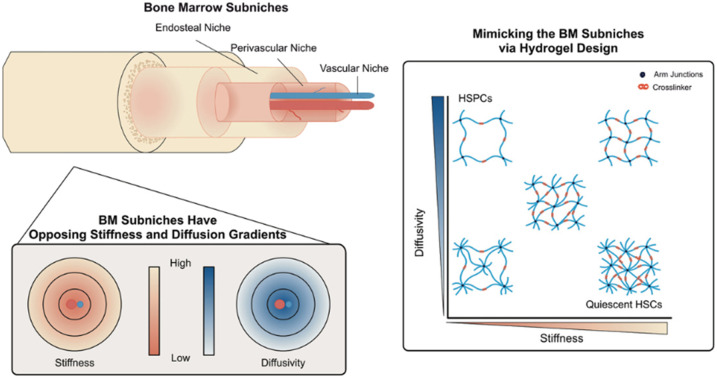

## Introduction

Hydrogels are crosslinked macromolecular systems (networks) that may have inherent properties of moderate or high swelling agent retention, network flexibility, and tunability of mechanical properties.^1,2^ In addition, they may exhibit biocompatibility, which renders them desirable carriers for biomedical applications.^3,4^ These may include their use as wound dressings, contact and intraocular lenses, drug delivery carriers, and tissue engineering scaffolds.^3,5,6^ Especially in scaffold applications in tissue engineering, researchers can exploit the highly tunable ability of hydrogels to engineer them as tools to mimic the properties of the extracellular matrix (ECM) of their intrinsic niche for *in vitro* 3D culture.^7^

Tuning the properties of hydrogel systems for applied cell use begins with the material of choice, whether derived, synthetic, or a combination of the two.^1^ Natural materials such as alginate, hyaluronic acid, and collagen are found in the native extracellular matrix (ECM) and can form hydrogels that are readily enzymatically degradable and contain cell-specific binding motifs.^8,9^ Synthetic polymers such as polyacrylamides, poly(vinyl alcohol), and poly(ethylene glycol) (PEG) do not inherently provide cell-specific biochemical cues; however, they offer researchers precise control over the mechanical and structural properties of resulting hydrogels.^3,9^ Using polyaddition polymerization, polymers can be synthesized with high structural fidelity, including defined arm length and specific multi-arm functionality.^9^ Among these, synthetic polymer PEG-based systems have been widely used for various cell-based applications and were therefore selected for this study.^10–14^

PEG-based hydrogels offer hydrophilicity, relative chemical inertness, and biocompatibility. PEG can be selectively modified for a myriad of properties, including end-group functionalization, molecular weight, and number of arms.^15–17^ Commercially available PEG polymers functionalized with end groups such as hydroxyls (PEG-OH) and amines (PEG-NH_2_) can be further modified with different functional groups such as norbornene (PEGNB) for downstream applications or functionalized with binding motifs, ligands, *etc*., as well as peptide crosslinkers that make the system more biocompatible for use with specific cell types or better able to mimic the properties of different tissues and organs.^18,19^ PEG is therefore uniquely suitable for generating libraries of hydrogels with tunable properties, which is the focus of this work.

In recent years, researchers have sought to understand how the stiffness of the BM microenvironment influences BM cell behavior using physically tuned lab-synthesized hydrogel systems. The BM microenvironment spans a wide stiffness range (0.1–45 kPa), yet most studies focus on mid-range stiffness values (∼20 kPa).^20–22^ For example, Shi *et al.* explored hydrogel stiffness alone, modulating the elastic modulus from 0.5 to 30 kPa and found that increased matrix stiffness inhibited HSC quiescence and favored myeloid lineage commitment.^22^ While much research has focused on stiffness effects, growing evidence suggests that solute diffusion is equally important.^23–25^

Notably, Xia *et al.*^26^ indicated that mesh size, distinct from stiffness, affects BM cell behavior. Using a polyacrylamide hydrogel-on-glass system with a constant stiffness of ∼15 kPa but varying mesh sizes, they showed that mesh size significantly influences the mechanobiology of human bone marrow mesenchymal stem cells (hBMSCs).

Jansen *et al.*^18^ showed that diffusivity impacts paracrine signaling within PEG hydrogels and demonstrated that reduced solute transport limited cytokine availability, thereby altering stem cell fate decisions. Although PEG-based hydrogels have previously been employed to mimic the BM microenvironment, there has yet to be a comprehensive study that independently varies mesh size, solute diffusivity, and stiffness.^14,18,27–30^ However, mimicking both BM stiffness and solute diffusion properties and decoupling these factors will be essential for advancing next-generation stem cell culture systems. Such efforts could better support precise control over HSC renewal, differentiation, and function. Moreover, these insights may also extend to optimizing culture conditions for other stem cell types.

To address this need and provide a rationally designed hydrogel system, we created a library of fully synthetic 3D *in vitro* hydrogel culture systems that control stiffness and diffusivity. We achieved this by varying the polymer density, number of arms, and chain length of PEG within the biologic conditions of the bone marrow niche. It should be noted that our earlier work focused on synthesizing polymer networks using a nondegradable crosslinker, which impedes a cell's ability to degrade and modulate its environment.^9,19,26^ In this work, we have created a library of MMP-sensitive degradable PEGNB hydrogels that span a physiologically relevant range of BM-like stiffnesses and diffusivities. Mechanical characterization confirmed that these hydrogels provide a vast range of swelling potentials, stiffness, and diffusivities. Finally, we validated the ability of these peptide-crosslinked hydrogel systems to support the three-dimensional (3D) culture of several cell types, including induced pluripotent stem cells (iPSCs) and primary hematopoietic stem cells. Encapsulated cells remained viable for 7 days, demonstrating the potential for these hydrogels to culture stem cells. This platform offers a powerful tool to better understand the mechanobiology and response of stem cells to a range of stiffnesses and diffusivities.

## Materials and methods

### Poly(ethylene glycol)-norbornene (PEGNB) functionalization

**Fig. 1:**
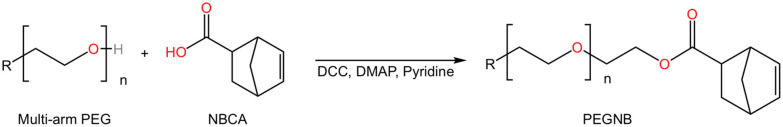
Synthesis reaction scheme of PEGNB hydrogels.

Six formulations of multi-arm hydroxyl-terminated PEG (PEG-OH) precursor polymers (4-arm 10 kDa, 4-arm 20 kDa, 6-arm 15 kDa, 6-arm 30 kDa, 8-arm 20 kDa and 8-arm 40 kDa custom synthesized) (JenKem Technology; Plano, TX) were chosen to explore the relationship of the number of arms (4, 6, or 8) and the chain length (number of repeated units, *N*_j_) on the stiffness and diffusivity of the synthesized hydrogel systems. PEG (PEG-OH) precursor polymers were norbornene (NB)-functionalized to create PEG-NB, as shown in Fig. 1.

Norbornene functionalization was achieved by the reaction of PEG-OH and 5-norbornene-2-carboxylic acid (NBCA) (Sigma-Aldrich; St Louis, MO) *via N*,*N*′-dicyclohexylcarbodiimide (DCC) (Sigma-Aldrich; St Louis, MO) coupling as previously described by Richbourg and Peppas.^31^ In relation to PEG-OH terminations, 5× molar equivalents of DCC were dissolved in dichloromethane (DCM) (Sigma-Aldrich; St Louis, MO) under a nitrogen atmosphere, stirring at 400 rpm. After 10 minutes of purging, 10× NBCA was added dropwise to the flask and left to react for 25 minutes, under a nitrogen atmosphere. Shortly after the addition of the NBCA, the solution became white and opaque, indicating the formation of the by-product dicyclohexylurea and the intermediate dinorbornene carboxylic acid anhydride. After reacting, the remaining solution was centrifuged at 3000 rpm at room temperature for 10 minutes. This led to the separation of a white, opaque by-product in the supernatant from the dinorbornene carboxylic acid anhydride underneath. The dinorbornene carboxylic acid anhydride was extracted and then added to 5 g of multi-arm PEG that was pre-mixed with 0.5× 4-(dimethylamino)pyridine (DMAP) (Sigma-Aldrich; St Louis, MO) and 5× pyridine, all dissolved in DCM under nitrogen purging and on ice. After 25 minutes, the nitrogen line was turned off, and the reaction mixture was left to react on ice overnight and filtered the next day.

The filtered solution was precipitated in ice-cold diethyl ether and centrifuged at 4000 rpm at 0 °C for 20 minutes. The supernatant was removed and replaced with fresh ice-cold diethyl ether. The PEGNB pellet was resuspended in the diethyl ether before another round of centrifugation. The supernatant was removed, and the pellet was left uncovered in air under a fume hood to allow excess diethyl ether to evaporate off overnight. Representative molecular structures of the norbornene-terminated multi-arm PEG formulations are shown in SI Fig. S1.

The dry pellet was resuspended and homogenized in DI water, dialyzed against DI water for at least 24 hours (MW 2000) with at least three water changes, lyophilized, and stored at −80 °C until use. Functionalization was confirmed *via*^1^H-NMR (CDCl_3_): *δ*-6.2–6.05 ppm (norbornene: 2H, –CH=CH–) and 3.68–3.6 ppm (PEG backbone: 4H, –CH_2_–CH_2_–O–) (SI Fig. S2).

### Synthesis of PEGNB hydrogels

Polymerization was achieved through thiol–ene photocrosslinking. PEGNB (SF. 1, Fig. 1) was dissolved in PBS (pH 7.4) and crosslinked using either dithiothreitol (DTT) or the degradable peptide crosslinker KCGPQG↓IWGQCK. Crosslinkers were added at a 2 norbornene : 1 crosslinker molar ratio relative to the theoretical maximum norbornene functionality to account for incomplete PEGNB functionalization and promote complete gelation. Lithium phenyl-2,4,6-trimethylbenzoylphosphinate (LAP) (Sigma-Aldrich; St Louis, MO) was used as the photoinitiator (0.5 mM).

The multi-arm PEG solutions were poured into a short plate hydrogel setup with a 1.5 mm spacer (Bio-Rad) and exposed to UV light (365 nm, 20 mW cm^−2^) for 2 min on each side on a UV transilluminator plate (UVP Analytik Jena). Hydrogel discs of 10 mm or 5 mm diameter were punched out of the sheets using a circular biopsy punch for further characterization.

### Swelling

Swelling volumes were determined using the buoyancy-based method of swelling reported in numerous previous Peppas publications and https://www.hydrogeldesign.org.^1,2,15,32,33^ The 10 mm diameter hydrogel discs were weighed at three different states – relaxed, swollen, and dry – in both air (*m*) and under a nonsolvent (*m*′) using a density kit setup (Mettler Toledo, Columbus, OH). The weight was measured immediately after polymerization, after complete swelling in DI water for 24 hours, and after complete drying at 50 °C under a vacuum. The volume (*V*) of the hydrogel was calculated using this equation: 
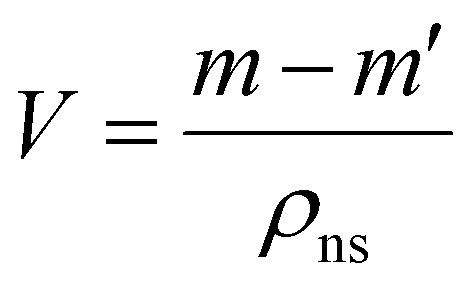
, where *ρ*_ns_ is the density of the nonsolvent hexanes (*ρ*_ns_ = 0.66 gmL^−1^). The polymer volume fractions at the relaxed states (*υ*_2,r_) and at the swollen state (*υ*_2,s_) were calculated using the equations 
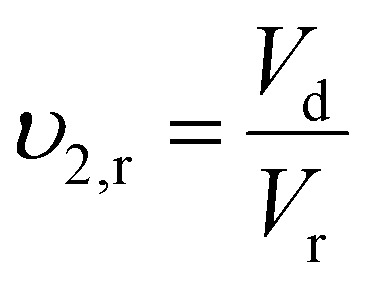
 and 
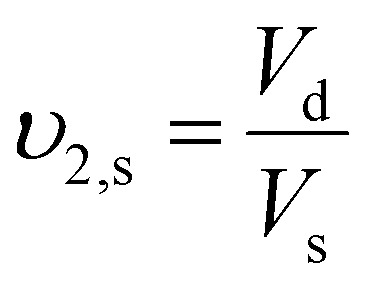
, respectively.

The reference ratio (*θ*) was determined using the ratio of the hydrogel in the swollen state to the relaxed state: 
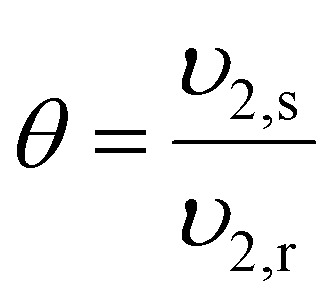
.

### Approximate mesh size

From the relaxed and swelling polymer fractions from the swelling assay, the average molecular weight between crosslinks (*M*_c_) was calculated using the Peppas–Merrill equation (eqn (1)) and the mesh size was subsequently calculated using the Peppas–Barr–Howell equation (eqn (2)).^34^ The results are found in the SI (SI Fig. S3).1
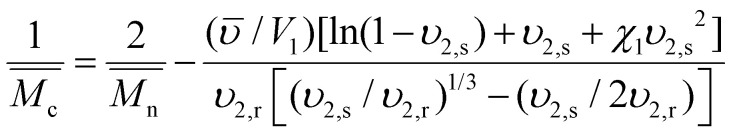


The Peppas–Merrill equation was used for calculating the average molecular weight between crosslinks 
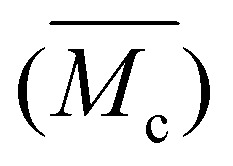
, where the molecular weight of linear polymer chains 
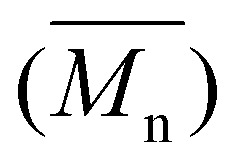
 is calculated by multiplying the MW of PEG repeat units (44 g mol^−1^) by the number of repeat units specific to each formulation, the specific volume of the polymer 
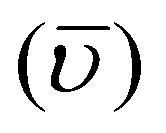
 is 1.124 g cm^−3^, the molar volume of the solvent (*V*_1_) is 18.1 cm^3^ mol^−1^, the relaxed polymer volume fraction (*υ*_2,r_) and swollen polymer fraction (*υ*_2,s_) are determined by the swelling studies, and the Flory interaction parameter (*χ*_1_) is 0.426.^35^ It must be noted that the following equation is only approximate as the term 3 indicates 3 bonds per PEG unit, but the bonds are not of the same size.2
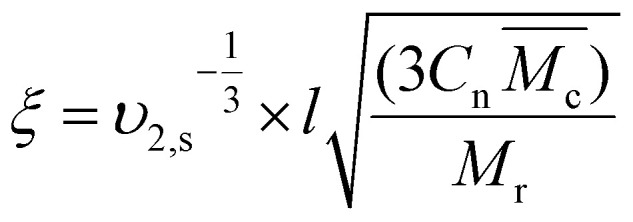


The approximate mesh size equation was used, where the average bond distance of PEG (*l*) is 1.54 Å, the characteristic ratio for PEG (*C*_n_) is 3.8,^35^ and the MW of PEG repeat units (*M*_r_) is 44 g mol^−1^.

### Stiffness

Stiffness was measured using a rheological test using a TA Instruments Discovery HR-3 Hybrid Rheometer (Malvern Instruments, UK). The rheometer was fitted with an upper 10 mm sandblasted Peltier plate and a lower 25 mm sandblasted plate. The hydrogels were centered between the two plates and subjected to an oscillatory sweep from 0.1 to 1% at a frequency of 1 Hz. The storage modulus (*G*′) was calculated at each point of the sweep. *G*′ is sampled at six points along the duration of the experiment and calculated using the rheometer's software.

### Fluorescence recovery after photobleaching (FRAP)

We used a Zeiss 710 LSM confocal microscope for fluorescence recovery after photobleaching using a ×20 objective at ×2 zoom (NA = 0.8, WD = 0.55 mm, pinhole = 1.00 AU) (Zeiss, Germany). We diffused fluorescein (FL00) (Sigma-Aldrich; St Louis, MO) and a 4 kDa FITC-dextran (FD4) (Sigma-Aldrich; St Louis, MO) probe solution into 5 mm diameter discs for 24 hours. We took 3 images per disc at a 512 × 512-pixel resolution with a pixel size of 0.42 µm at 8% or 10% laser power for the FL00 and FD4, respectively. The hydrogels were imaged at a *z*-height of 750 µm from the bottom of the hydrogel. The hydrogels were exposed to 100% laser intensity (circular ROI-128 × 128 pixel) using a 488 nm argon laser after three pre-bleaching images had been taken. The recovery images were taken at a scan speed of 240 ms per frame with a 0.3-s delay between frames.

TIF files were analyzed using the FRAP analysis program reported by Jönsson *et al.*^36^ and modified by Richbourg^15^ that supports high-throughput analysis to produce diffusion coefficients and immobilized solute fractions.

### Induced pluripotent stem cell (iPSC) culture

Induced pluripotent stem cells (WiCells) were cultured in a 6-well plate using E8 complete media (Thermo Fisher Scientific, Waltham, MA). Medium changes were performed every day. When cells reached 80% confluence, cells were passaged using the single cell passaging technique. In brief, iPSCs were detached from the plate using Accutase, incubated at 37 °C for 5 minutes, and centrifuged at 300*g* for 5 minutes. The cells were resuspended in fresh media and passaged at a 1 : 20 dilution.

### Primary hematopoietic stem cell (HSC) culture

CD34+ hematopoietic stem cells (G-CSF-mobilized from bone marrow; StemCell Technologies, Vancouver, Canada) were cultured in 12-well plates using StemSpan SFEM II media supplemented with 50 ng mL^−1^ stem cell factor (SCF), 50 ng mL^−1^ thrombopoietin (TPO), 50 ng mL^−1^ Flk2/Flt3, 10 ng mL^−1^ Il-3, and 50 ng mL^−1^ Il-6 (StemCell Technologies, Vancouver, Canada). After seeding onto the culture plate, a full medium change was performed after 5 days, and subsequent medium changes were performed every 3 days. For the full medium change, the medium was removed from the well and then centrifuged at 300*g* for 5 minutes. The medium was carefully removed, so as not to disturb the cell pellet, and the cells were resuspended in fresh media and reintroduced to the wells.

### Cell encapsulation in hydrogels

Hydrogels were prepared as previously described with modifications for cell culture and adhesion to the system. E8 complete media and StemSpan SFEM II media were used as the solvents instead of PBS for iPSC and HSC encapsulation, respectively, and the cell-binding motif RGD (GCGYGRGDSPG) was added to encourage cell adhesion to the hydrogel scaffold. In brief, 125 µL of each hydrogel formulation was made. Lyophilized PEG and enzymatically degradable peptide (KCGPQG↓IWGQCK) or DTT were added to a final concentration as listed in the SI. RGD and LAP were added to the final concentrations of 2 mM and 0.5% (w/v), respectively. Finally, cells were spun down at 300*g* for 5 minutes, and the hydrogel solution was added. 50 µL of the hydrogel and cell solution were pipetted into each well of a µ-Slide 18 Well Glass Bottom (Ibidi, Fitchburg, WI) and exposed to 365 nm UV light at 10 mW cm^−2^ for 120 seconds. The hydrogels were fed with 100 µL of E8 complete or StemSpan media with the cytokines for iPSCs or HSCs, respectively. Medium changes were performed daily for iPSCs and every 3 days for HSCs.

### Cell viability

After 7 days of culture, cell viability was determined using a calcein green (1 : 1000)/ethidium homodimer-1 (1 : 500) LIVE/DEAD stain (ProteinTech, Rosemont, IL). Cells were stained in the dark at room temperature for 20 minutes. The dye solution was removed, and hydrogels were washed twice with DPBS, waiting 5 minutes between washes. Four 3D regions of interest for each hydrogel formulation were imaged using a Leica SP8 confocal microscope and analyzed using ImageJ to quantify the proportion of the volume of live to the volume of dead cells and averaged for each formulation.

### Statistical analysis

Measurements were performed with *N* = 15 replicates per condition for swelling and rheology experiments, and *N* = 9 replicates per condition with *n* = 3 images taken per hydrogel sample for FRAP experiments. All results were analyzed using a two-way ANOVA (3 or more experimental conditions), followed by Tukey's multiple comparison test, except cell viability, which was analyzed using one-way ANOVA and multiple unpaired *t*-tests for matched conditions using Prism 10 (GraphPad). Significance is denoted as follows: *p < 0.05, **p < 0.01, ***p < 0.001, and ****p < 0.0001. Data are presented as mean ± standard deviation.

## Results

### Consistent swelling trends across peptide- and DTT-crosslinked PEGNB hydrogels reveal predictable design rules

We first verified the chemical structures of multi-arm PEGNB modification by ^1^H-NMR (SI Fig. S1). More specifically, we determined that all formulations exhibited approximately the same norbornene modification (59–65%), when expressed as % modification, as shown in SI Table S1. This degree of substitution is sufficient to support robust and reproducible thiol–ene hydrogel crosslink formation across all formulations tested. Increased norbornene incorporation may additionally influence polymer hydration and solution behavior due to the comparatively hydrophobic nature of norbornene-containing segments.^37,38^ Next, a library of PEGNB hydrogels was formulated using three structural parameters – percent polymer volume (ppv), number of arms, and chain length (*N*_j_).

**Fig. 2:**
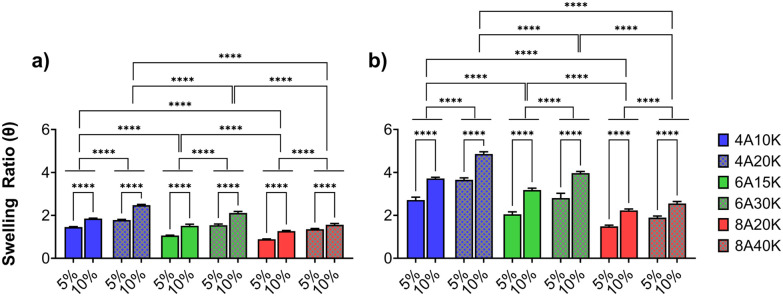
Swelling ratios for PEGNB hydrogels: (a) DTT-crosslinked and (b) peptide-crosslinked. By independently varying hydrogel parameters, increases in ppv and chain length correlated with higher swelling ratios. Conversely, increasing number of arms correlated with lower swelling ratios. The introduction of the degradable peptide resulted in marginally higher swelling ratios (*N* = 10–15). ****p < 0.0001.

To characterize swelling behavior, equilibrium swelling ratios were measured to quantify water uptake from the relaxed to fully swollen state (SI Tables S2 and S3). Across all formulations, swelling ratios ranged from 0.89 to 4.86, demonstrating substantial tunability based on hydrogel composition (Fig. 2). Fold-change comparisons between formulations are shown in SI Table S4.

Swelling trends were consistent across both DTT-crosslinked (Fig. 2a) and peptide-crosslinked (Fig. 2b) hydrogels. In both systems, increasing ppv from 5% to 10% resulted in increased swelling across all matched formulations (*e.g.* 5% 4A10K and 10% 4A10K). Similarly, increasing PEG chain length (*N*_j_) led to higher swelling ratios across all matched formulations (*e.g.* 5% 4A10K and 5% 4A20K). For example, within each arm group, longer chain length formulations (4A20K, 6A30K, and 8A40K) consistently exhibited greater swelling than their shorter chain length counterparts (4A10K, 6A15K, and 8A20K). In contrast, increasing the number of arms led to reduced swelling, with 4-arm hydrogels swelling the most and 8-arm hydrogels swelling the least across all conditions.

While these structure–property relationships were preserved, peptide-crosslinked hydrogels exhibited modestly higher swelling ratios compared to their DTT-crosslinked counterparts. This increase was consistent across all ppv conditions and macromer architectures, with peptide-crosslinked formulations reaching swelling ratios as high as 4.86 at 10% ppv, indicating that degradability shifts the magnitude of swelling without altering the governing trends.

Analysis of fold-changes further supported these observations (SI Table S4). Increasing *N*_j_ consistently increased swelling, while increasing arm number reduced swelling across both systems. The magnitude and direction of these fold-changes were comparable between DTT-crosslinked and peptide-crosslinked hydrogels, reinforcing that network architecture remains the dominant determinant of swelling behavior.

Taken together, these results demonstrate that PEGNB hydrogel swelling can be predictably and reproducibly tuned through variations in polymer concentration, PEG chain length, and macromer architecture.

### Hydrogel stiffness is modulated by polymer architecture and composition

**Fig. 3:**
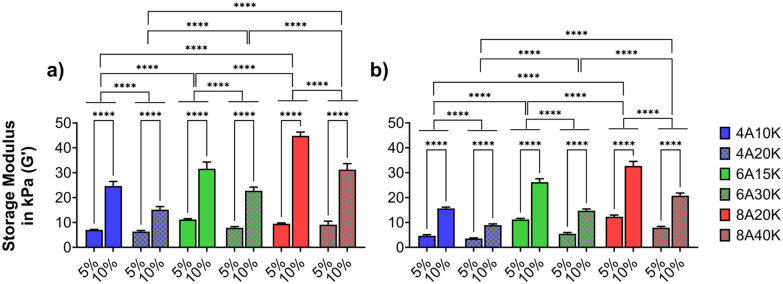
Storage modulus for PEGNB hydrogels: (a) DTT-crosslinked and (b) peptide-crosslinked. By independently varying hydrogel parameters, increases in ppv and the number of arms correlated with higher storage moduli. Conversely, increasing PEGNB chain length correlated with a lower storage modulus. The introduction of degradable peptides resulted in lower storage moduli across formulations (*N* = 10–15). ****p < 0.0001.

To evaluate the mechanical stiffness of the PEGNB hydrogels, oscillatory shear rheology was performed to measure the storage modulus (*G*′), a primary indicator of elastic behavior (Fig. 3). The fold-change comparisons for the DTT-crosslinked and peptide-crosslinked formulations were calculated (SI Table S5).

While stiffness can refer to various mechanical properties, in the context of soft synthetic hydrogels, stiffness can be best represented by the storage modulus (*G*′). In such systems, the loss modulus (*G*″) is extremely low; therefore, *G*′ was used as a direct readout of the hydrogel stiffness.

Across all formulations, stiffness values for DTT-crosslinked hydrogels (Fig. 3a) ranged from 6.4 kPa to 44.8 kPa, spanning physiologically relevant BM stiffnesses, including soft vascular niches (∼3 kPa) to stiffer bone marrow regions (∼44 kPa).^27^ These results confirm that PEGNB hydrogels can be engineered to recapitulate a broad mechanical spectrum.

Stiffness trends were consistent across both DTT-crosslinked (Fig. 3a) and peptide-crosslinked (Fig. 3b) hydrogels. Increasing ppv from 5% to 10% led to a significant increase in *G*′ across all formulations, reflecting increased network density. For example, the 4A10K formulation exhibited a substantial increase in stiffness with increasing ppv. Across all formulations, the magnitude of this increase varied, with the largest fold-change observed in the 8A20K system and the smallest in the 4A20K system.

PEG chain length (*N*_j_) had an inverse effect on stiffness. When ppv and arm number were held constant, increasing *N*_j_ resulted in decreased stiffness, consistent with reduced crosslink density in longer polymer chains. For example, the 5% 4A10K formulation exhibited higher stiffness than the 5% 4A20K formulation. This trend was preserved across both ppv conditions and all arm numbers.

In contrast, increasing the number of arms resulted in increased stiffness. At constant ppv and *N*_j_, hydrogels with a higher number of arms exhibited greater stiffness, consistent with increased crosslinking density. This trend was consistent across both DTT-crosslinked and peptide-crosslinked systems.

Although the overall trends were conserved, peptide-crosslinked hydrogels exhibited lower stiffness compared to their DTT-crosslinked counterparts. This reduction in *G*′ was consistent across all formulations and is likely due to the incorporation of the peptide crosslinker, which introduces additional flexibility and reduces effective network rigidity.^39^

Taken together, these results demonstrate that hydrogel stiffness can be systematically tuned through polymer concentration, chain length, and macromer architecture. These design principles are conserved across peptide-crosslinked and DTT-crosslinked systems, with degradability introducing a predictable reduction in stiffness without altering the underlying trends.

### Solute diffusivity is primarily governed by PEG chain length and crosslinker chemistry

**Fig. 4:**
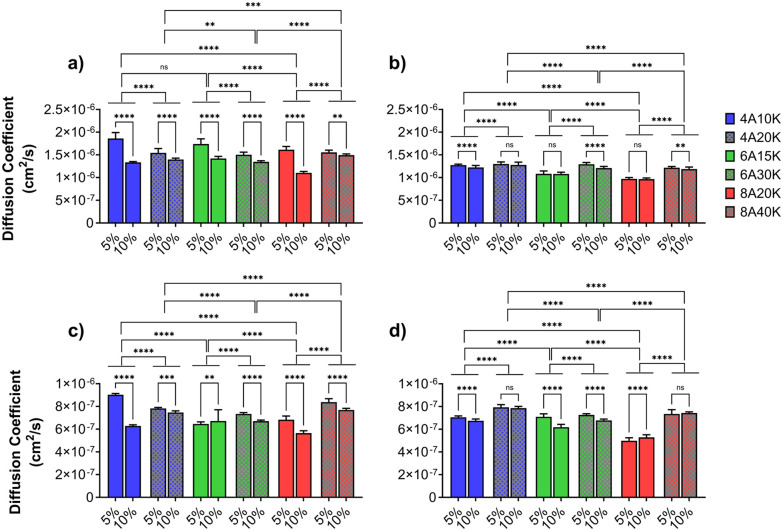
Diffusion coefficients for PEGNB hydrogels. (a and c) DTT-crosslinked and (b and d) peptide-crosslinked using fluorescein (a and b) or FITC-dextran (4 kDa) (c and d). By independently varying hydrogel parameters, increases in ppv and arm functionality resulted in decreased solute diffusivity within the hydrogel network. Increases in chain length generally increased solute diffusivity. The replacement of DTT with the peptide crosslinker, though larger in size, led to an overall decrease in the diffusion coefficient compared to DTT-crosslinked hydrogels (*N* = 6–9). **p < 0.01, ***p < 0.001, and ****p < 0.0001.

To evaluate molecular transport within PEGNB hydrogels, fluorescence recovery after photobleaching (FRAP) was performed using two fluorescent probes: fluorescein (300 Da) (Fig. 4a and b) and FITC-dextran (4 kDa) (Fig. 4c and d). These were selected as representative model probes to characterize steric transport across formulations; while larger biomolecules are also physiologically relevant, the chosen probes provide a controlled basis for comparing network-dependent diffusion behaviour, specifically in denser hydrogels such as the one tested here.^39,40^ Calculated diffusivity values are shown in Fig. 4 and SI Table S8, with fold-change analyses provided in SI Tables S9–S13. The specific diffusion coefficient values reported in this section are obtained from fluorescein transport studies.

For DTT-crosslinked hydrogels (Fig. 4a and c), increasing ppv from 5% to 10% led to a consistent decrease in diffusivity for both probes, indicating that increased polymer volume results in a denser network that restricts solute transport.

For fluorescein, as the percent polymer volume increased from 5% to 10%, the diffusivity decreased (*e.g.* 1.86 × 10^−6^–1.5 × 10^−6^ cm^2^ s^−1^ from 5% 4A10K to 10% 4A10K, respectively). A similar trend was observed for FITC-dextran, where diffusivity was reduced at higher ppv.

Chain length (*N*_j_) had a more pronounced effect on diffusivity than ppv. Increasing *N*_j_ generally resulted in increased diffusivity across both probe sizes, consistent with larger network mesh sizes arising from longer polymer chains. This trend was observed across all arm numbers and ppv conditions.

The number of PEG arms also influenced diffusivity, though to a lesser extent than *N*_j_. Increasing PEG arm functionality generally decreased diffusivity, consistent with increased effective crosslink density and reduced network mesh size.

In peptide-crosslinked hydrogels (Fig. 4b and d), diffusivity was consistently lower compared to their DTT-crosslinked counterparts across all formulations and probe sizes. This suggests that the incorporation of degradable peptide-crosslinkers alters the network structure, potentially introducing transient interactions or steric hindrance that reduce effective solute transport. Despite this shift in magnitude, the relative trends with respect to ppv, *N*_j_, and arm number were preserved.

Taken together, these results indicate that solute diffusivity in PEGNB hydrogels is primarily governed by network density and polymer chain length, with degradability introducing an additional constraint that reduces overall transport without altering the underlying polymer structure–property relationships.

### Cell viability across cell types and hydrogel formulations shows high viability for *in vitro* 3D cell culture

To evaluate cytocompatibility, cells were encapsulated within PEGNB formulations spanning a range of mechanical and transport properties. The formulations tested included 5% 4A20K and 5% 8A40K, evaluated in both DTT-crosslinked and peptide-crosslinked hydrogels. These formulations were intentionally selected relevant to the BM niches, with stiffness ranging from ∼3.55 to 24.6 kPa and diffusivities from 1.21 × 10^−6^ to 1.86 × 10^−6^ cm^2^ s^−1^, reflecting variations across distinct BM subniches. Low stiffness and more permissive hydrogels were prioritized for iPSC encapsulation due to the known sensitivity of pluripotent stem cells to mechanical cues, whereas HSCs were evaluated across multiple formulations spanning broader mechanical and transport conditions relevant to perivascular and endosteal marrow niches. The goal of these studies was to establish cytocompatibility across representative BM-like environments rather than to comprehensively optimize each cell type across all hydrogel formulations.

**Fig. 5:**
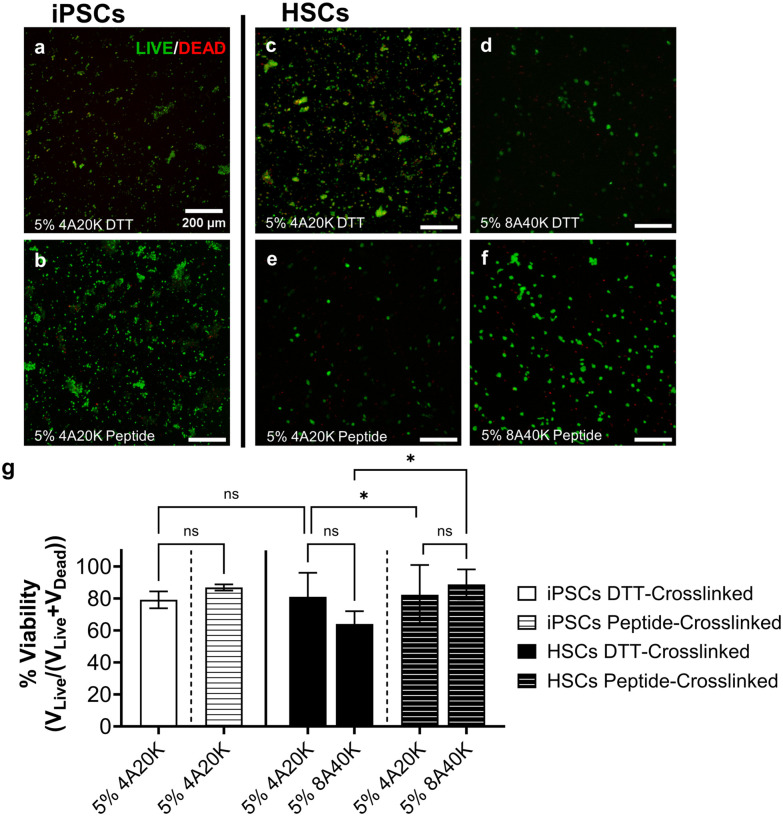
Stem cell viability in DTT-crosslinked (top row) and peptide-crosslinked (bottom row) PEGNB hydrogels show high cell compatibility across formulations. Representative live/dead images of iPSCs encapsulated in (a) 5% 4A20K DTT-crosslinked hydrogels (79% viability) and (b) 5% 4A20K peptide-crosslinked hydrogels (89% viability); representative live/dead images of primary HSCs encapsulated in (c) 5% 4A20K (81% viability) and (d) 5% 8A40K (64% viability) DTT-crosslinked hydrogels and (e) 5% 4A20K (82% viability) and (f) 5% 8A40K (89% viability) peptide-crosslinked hydrogels. (g) Quantitative viability analysis was performed across multiple fields of view and shows the results of these viability studies (iPSCs: *N* = 8, HSCs: *N* = 12); *p < 0.05. For all figures, the scale bar is 200 μm.

Viability was assessed after 7 days post-encapsulation using quantitative viability measurements from multiple fields of view across independent hydrogel replicates, providing a representative assessment of overall cell viability (Fig. 5).

Across all conditions, high cell viability was maintained, demonstrating that PEGNB hydrogels support cell survival across a range of mechanical and structural environments. iPSCs remained viable in both DTT-crosslinked and peptide-crosslinked 5% 4A20K formulations (79% and 89% viability, respectively), despite their sensitivity to mechanical stiffness.

HSCs exhibited robust viability across multiple formulations, including both peptide-crosslinked and DTT-crosslinked systems. In 5% 4A20K hydrogels, viability remained high in both DTT-crosslinked and peptide-crosslinked conditions (81% and 82%, respectively).

In 5% 8A40K hydrogels, DTT-crosslinked formulations showed a lower viability (64%), whereas introducing the degradable peptide crosslinker led to improved viability (89%), suggesting that increased mesh size and degradability may enhance cellular tolerance and distribution.

Overall viability was maintained across formulations, indicating that variations in the hydrogel structure did not substantially compromise cell survival and demonstrating the broad biocompatibility of PEGNB hydrogels for 3D cell culture. Notably, the peptide-crosslinked 5% 8A40K hydrogel supported the highest viability, consistent with the possibility that peptide-crosslinked matrices may better facilitate nutrient transport and cell–matrix remodeling. These results further suggest that peptide-crosslinked systems may provide advantages for supporting cell viability while maintaining tunable physical properties, although representative image appearance may vary due to local spatial heterogeneity within the hydrogels.

## Discussion

The goal of this study was to design a series of tunable PEG-norbornene (PEGNB) hydrogel platforms with mechanical and transport properties relevant to the bone marrow niche. Our aim was to enable physiologically meaningful 3D stem cell culture. To achieve this, we systematically varied three key network parameters – the initial polymer volume fraction in the gel, the number of arms, and the arm length of the PEGNB macromers. The goal was to evaluate how these variables can influence the hydrogel stiffness, volume swelling ratio, and solute diffusivity.

These formulations were synthesized under consistent conditions and crosslinked with either DTT, which is a small, nondegradable molecule, or with a matrix metalloproteinase (MMP)-degradable peptide, to investigate the influence of both permanent and cell-responsive network crosslinks. This work builds on our previously published mathematical modeling work by Richbourg and Peppas,^15^ which demonstrated the ability to decouple stiffness and diffusivity by manipulating hydrogel architecture. Here, we expand upon those insights by experimentally creating a library of PEGNB hydrogels and introducing a bioresponsive crosslinker. The MMP-sensitive peptide (KCGPQG↓IWGQCK) adds biological relevance for future cell culture applications by enabling dynamic matrix remodeling.

Hydrogel swelling behavior varied widely across formulations (Fig. 2), emphasizing the importance of the network structure in regulating swelling agent uptake. The extensive statistical significance observed across formulations (****p < 0.0001) indicates that the measured differences in the swelling ratio arise from deliberate hydrogel parameter tuning rather than experimental variability. Importantly, these structure–property relationships were conserved across both peptide-crosslinked and DTT-crosslinked systems, providing a robust framework for engineering hydrogel swelling behavior.

Increasing the number of PEGNB arms decreased the swelling ratio. This is attributed to the increased volume occupied by branched polymer chains, which limits the volume available for solvent uptake. As the volume of polymer chains increases, the solvent-accessible volume decreases, thus reducing the swelling ratio. These findings align with scaling theories such as those by de Gennes and Flory–Rehner, which account for polymer conformation and network constraints in three dimensions.^40,41^ Moreover, increasing the PEGNB arm length was found to increase swelling, due to the presence of uncrosslinked internal chain ends that do not contribute to network stiffness but increase free volume and contribute to swelling.^40,42,43^

**Fig. 6:**
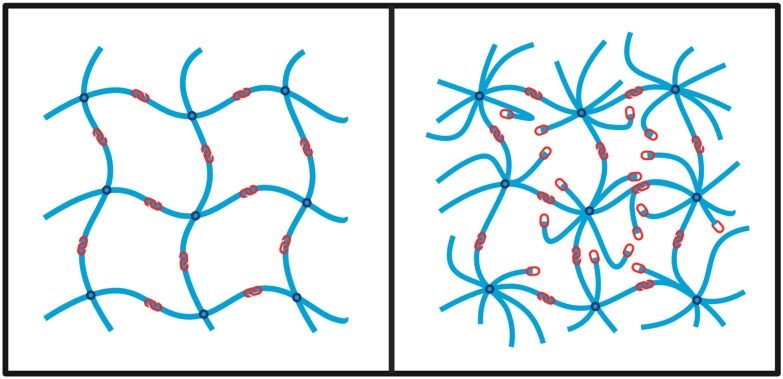
Schematic representation of a crosslinked network with (right) and without (left) pendant chains. These pendant chains may contribute to swelling without contributing to overall stiffness and crosslinking density.

We differentiate between external (usually chemical) crosslinks, which define the hydrogel's network structure, and internal crosslinks, which arise from PEG chains that do not participate in effective junction formation. The formulations used in the present study showed norbornene modification from 59% to 65%, resulting in a frequency of end-chain defects (*γ*) of about 0.4. The hydrogel formulations showed robust ability to form crosslinks consistently when exposed to covalent crosslinkers and a photoinitiator.^15,44,45^ While these defects are often overlooked, our data show that they significantly influence hydrogel swelling (Fig. 2). Formulations with a higher arm number and shorter arm lengths formed denser internal networks, limiting swelling despite constant nominal crosslink density. Our results show that hydrogel networks exhibit considerable heterogeneity, particularly with multifunctional monomers. For example, the increase in swelling seen with longer PEGNB arms potentially reflects the presence of pendant chains that act as internal plasticizers rather than contributing to stiffness (Fig. 6).^40,42,46^

From the swelling data (SI Fig. S3), we were able to calculate the estimated mesh size (∼5.5–14.2 nm) and radius (∼2.3–13.8 nm). Our data coincide with previously reported mesh sizes of similar multi-arm PEG hydrogel systems.^41,47–49^ Rehmenn *et al.* reported mesh sizes for 4-arm PEGNB systems of varying percent polymer volumes (4%, 10%, and 20%) and molecular weights, which correspond to our reported chain lengths (5 kDa, 10 kDa, and 20 kDa) (∼5 to 15 nm).^48^ Their formulations show similar governing trends: increases in ppv lead to decreases in mesh size, but increases in chain length lead to increases in mesh size.^48,49^

Rheological measurements led to the identification of several trends. First, stiffness increased with a higher ppv and decreased with longer arm lengths or lower arm numbers. Doubling ppv from 5% to 10% led to a 1.4- to 3.7-fold increase in shear modulus, consistent with classical rubber elasticity theory.^41^ Our experimental stiffness values were significantly lower than those predicted by Richbourg and Peppas,^15^ but they accounted for end-chain defects up to 0.25, whereas our polymers were determined to be about 0.4. This discrepancy likely reflects the influence of internal defects and dangling chain ends, factors that are often overlooked in theoretical treatments but were carefully considered in the present structural analysis. The high degree of statistical significance between formulations supports the robustness of the observed mechanical trends and demonstrates that hydrogel stiffness can be systematically tuned through independent variation of polymer parameters. These findings reinforce the conclusion that arm functionality, chain length, and ppv each contribute predictably to network mechanics.

The prevalence of highly significant comparisons (****p < 0.0001) indicates that changes in the storage modulus were strongly formulation-dependent, validating the ability to decouple and reproducibly control hydrogel mechanical properties through polymer design.

Diffusion studies showed that the apparent solute diffusion coefficients of our hydrogel formulations fall within an order of magnitude of the diffusivity reported for small molecules in bone marrow, such as fluorescein (∼4.5 × 10^−6^ cm^2^ s^−1^).^48^ While not directly comparable to the apparent diffusion coefficient of water (*D* ≈ 23 × 10^−6^ cm^2^ s^−1^), our diffusion values suggest that the designed PEGNB hydrogels are appropriate analogs for mimicking native marrow transport properties.

As expected, higher swelling generally corresponded to increased diffusivity, due to the enlarged mesh size. The statistically significant differences in diffusivity across formulations confirm that solute transport is strongly governed by hydrogel network architecture rather than random experimental variation. In particular, the repeated observation of highly significant comparisons (****p < 0.0001) supports the conclusion that ppv, arm functionality, and PEG chain length independently regulate molecular transport behavior within PEGNB hydrogels.

These findings validate the utility of our hydrogel library in modeling microenvironments where mass transport is a key parameter.^15,18,26,46^

Collectively, our data on the influence of network structure on hydrogel properties is supported by previous studies. Specifically, increasing arm number enhances crosslinking density, thereby increasing stiffness while reducing swelling and diffusivity. In contrast, longer arms reduced junctional density, leading to lower modulus hydrogels with increased swelling and diffusivity. Increasing the polymer volume fraction also leads to a higher storage modulus and lower swelling and diffusivity by increasing the density of reactive groups. Lee *et al.* reported similar findings while similarly varying parameters of PEG hydrogels.^39,50,51^ Using linear PEG hydrogels, they varied parameters, including molecular weight (2, 3, 4, and 10 kDa) and polymer concentration (10%, 15%, and 20%). Additionally, they also included an 8-arm 10 kDa PEGNB formulation, although they did not explicitly compare this formulation with the linear PEG hydrogels.

For all formulations, regardless of structural arm differences, they also reported increased stiffness with increased PEG concentration and decreased PEG molecular weight. Importantly, our data fill current gaps in the literature, which include a predominant focus on DTT-crosslinked systems or models that assume ideal network formation, which overlook the complexities of real hydrogel architectures. Few studies have systematically evaluated the combined effects of arm number, arm length, and ppv in peptide-crosslinked PEGNB hydrogels, particularly those incorporating MMP-sensitive crosslinkers. Moreover, theoretical predictions often neglect internal network defects such as loop formation, chain-end defects, and polymer entanglements, despite their significant impact on mechanical and transport properties.^42^ Prior work by Rezakhani and Lutolf demonstrated that reducing loop defects can decrease swelling in multi-arm PEG systems, suggesting that network defects may influence hydrogel expansion at low polymer concentrations.^50^ While increasing polymer concentration would generally be expected to reduce intramolecular loop formation, differences in network defect prevalence between 4-arm and 8-arm PEGNB formulations may partially contribute to the higher swelling observed in the 4-arm gels. However, we believe the dominant effect in our system is still likely related to differences in polymer architecture and solvent-accessible volume.^50–53^

Mechanical testing studies demonstrate that the use of an MMP-degradable crosslinker in the hydrogels further influenced these properties. Hydrogels formed with the peptide crosslinker showed higher swelling compared to those with DTT, consistent with the peptide's larger molecular weight (∼1300 Da *vs.* 154 Da) and resulting in a lower effective crosslinking density (Fig. 4a and b). Importantly, the relative trends in stiffness and diffusivity remained consistent across peptide-crosslinked and DTT-crosslinked networks, indicating that the structural insights drawn here are broadly applicable.

Finally, cell viability was evaluated using iPSCs (Fig. 5a and b), which are highly sensitive to mechanical cues, and primary HSCs (Fig. 5c–f), which reside within bone marrow niches and represent the target cell population for this system. From the library of hydrogels studied, we selected four formulations that fell within the physiologically relevant range of the bone marrow microenvironment: 10% 4A10K DTT-crosslinked, 5% 4A20K DTT-crosslinked and peptide-crosslinked, 10% 4A20K peptide-crosslinked, and 5% 8A40K DTT-crosslinked and peptide-crosslinked.

The endosteal niche, closest to the bone surface, has a stiffness of around 35 kPa and is a site where quiescent HSCs are found.^27^ The perivasculature niches have a lower stiffness, lower than 10 kPa, where HSC differentiation into blood and immune system components occurs. Endothelial progenitor cells prefer a substrate between these two values (∼10 kPa), and as previously stated, play a crucial role in vascular repair and formation.^7,21,54,55^ The selected hydrogel formulations spanned a stiffness range from ∼3.55 kPa (low stiffness) to ∼24.6 kPa (medium-to-high stiffness) and a diffusivity range from 1.21 × 10^−6^ cm^2^ s^−1^ to 1.86 × 10^−6^ cm^2^ s^−1^. Notably, three of the selected formulations had similar diffusivities (∼1.21 × 10^−6^ cm^2^ s^−1^) despite substantial differences in stiffness (Fig. 4). This supports the hypothesis that stiffness and diffusivity can be decoupled through deliberate control of network architecture.

Importantly, we found that cell viability remained high (Fig. 5) in these selected formulations, indicating their suitability for future cell culture studies. This confirms the compatibility of these hydrogels with encapsulated cells, thus highlighting the potential of this platform for supporting mechanistic investigations into how decoupling stiffness and diffusivity impacts HSC maintenance and differentiation, an area that will be explored in future studies.

## Conclusions

By systematically varying network architecture, specifically polymer volume fraction, arm number, and arm length, we produced a family of tunable hydrogels that spans a physiologically relevant range of stiffness, swelling, and diffusivity suitable for bone marrow mimetic applications. In this work, we demonstrated the effect of internal chemical and physical crosslinks and tie-points and uncrosslinked tethered ends on their swelling behavior and mechanical properties. We validated key structure–function predictions. We addressed prior limitations and incorporated bioresponsive degradation to allow cell-mediated remodeling. These findings provide a robust framework for the rational design of hydrogels tailored to ECM characteristics critical for hematopoietic stem cell maintenance and function.

## Author contributions

Kristie Cheng: data curation, formal analysis, investigation, visualization, software, writing – original draft, and writing – review and editing. Evelyn Lim: data curation. Brett Stern: investigation and data curation. Janet Zoldan and Nicholas Peppas: conceptualization, funding acquisition, resources, project administration, supervision, writing – original draft, and writing – review and editing.

## Conflicts of interest

We, the authors of this manuscript, do not declare any financial or ethical conflicts of interest.

## Supplementary Material

BM-014-D6BM00440G-s001

## Data Availability

The data supporting this article have been included as part of the supplementary information (SI). Supplementary information: H^1^-NMR spectra, further experimental details and data, and Tables S1–S13. See DOI: https://doi.org/10.1039/d6bm00440g.
