# Supplementary material for: Degradable multi-arm PEG hydrogels with tunable stiffness and diffusivity
Source: Biomater Sci. 2026 Jun 17;14(14):3874–84. doi: 10.1039/d6bm00440g (PMC13273626; doi:10.1039/d6bm00440g)
Supplement: BM-014-D6BM00440G-s001 [file BM-014-D6BM00440G-s001.pdf]

Supplementary Information (SI) for Biomaterials Science.
This journal is © The Royal Society of Chemistry 2026

Degradable Multi-Arm PEG Hydrogels with Tunable Stiffness and Diffusivity

Kristie Cheng[a,c], Evelyn Lim[a,c], Brett Stern[a], Janet Zoldan[a]†, Nicholas Peppas[a,b,c,d,e,f]†,

—

* Corresponding author. E-mail address: zjanet@utexas.edu (J. Zoldan), peppas@che.utexas.edu (N. Peppas).

[a.] Department of Biomedical Engineering, The University of Texas at Austin, 107 W Dean Keeton Street, Austin, TX 78712, United States
[b.] McKetta Department of Chemical Engineering, The University of Texas at Austin, Austin, Texas, USA
[c.] Institute for Biomaterials, Drug Delivery, and Regenerative Medicine, The University of Texas at Austin, Texas, USA
[d.] Department of Pediatrics, Dell Medical School, 1400 Barbara Jordan Blvd., Austin, TX, USA
[e.] Department of Surgery and Perioperative Care, Dell Medical School, The University of Texas at Austin, Austin, TX, USA
[f.] Division of Molecular Pharmaceutics and Drug Delivery, College of Pharmacy, The University of Texas at Austin, Austin TX, USA
† Corresponding author. E-mail address: zjanet@utexas.edu (J. Zoldan), peppas@che.utexas.edu (N. Peppas).

Supplementary Results:

Mesh size:

The mesh size (ξ) of the PEGNB hydrogels was estimated using the Peppas-Merril equation, which incorporates volume fraction (ppv), average molecular weight between crosslinks (Mc), and swelling ratio. This calculation provides an indirect assessment of the network structure, as the mesh size was not directly measured. It is important these values represent theoretical estimates and were omitted in prior related studies. To further contextualize diffusion behavior in these hydrogels, we also evaluated the mesh radius (r_m), which incorporated the geometry of the multi-arm PEGNB repeating unit as a refinement of mesh size. Established by Richbourg and Peppas, mesh radius is a more encompassing measure of the area unobstructed by polymer fibers than mesh size as it accounts for the arm-functionality of the polymer which has a different chemical structure, and thus different mesh orientation when crosslinked compared to a linear polymer system (Richbourg et al., 2021). The transform of the mesh size to mesh radius ($\frac{\sqrt{6}}{3}\xi\ , f = 4$; $\frac{1}{2}\xi\ , f = 6\ ,\&\ \frac{\sqrt{2}}{4}\xi\ , f = 8$) offers insight into how molecules may diffuse within the hydrogel in the absence of external forces. The calculated mesh sizes and mesh radii are presented in SF2. Notably, the mesh radiuses fall within the bounds of an ideal range for 3D cell encapsulation (~40 to 200 nm), enabling cell entrapment while maintaining sufficient porosity for solute exchange.

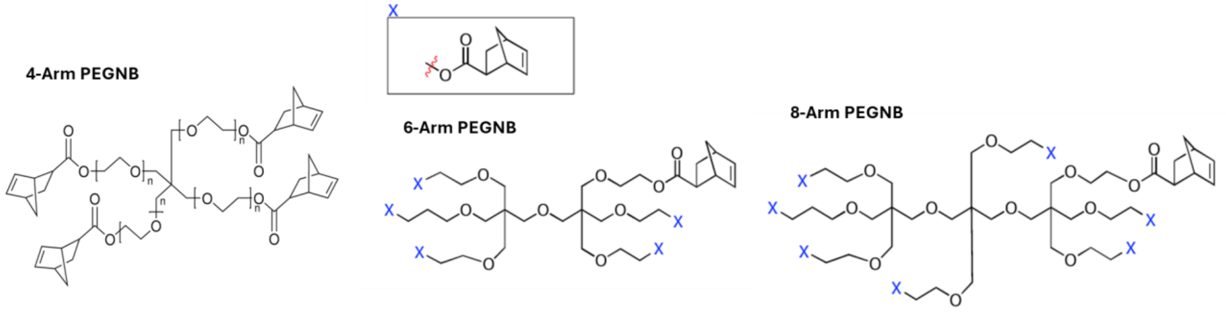

Supplementary Figure 1. Structure of multi-arm PEGNB polymers, where X denotes orthogonal norbornene functional groups.

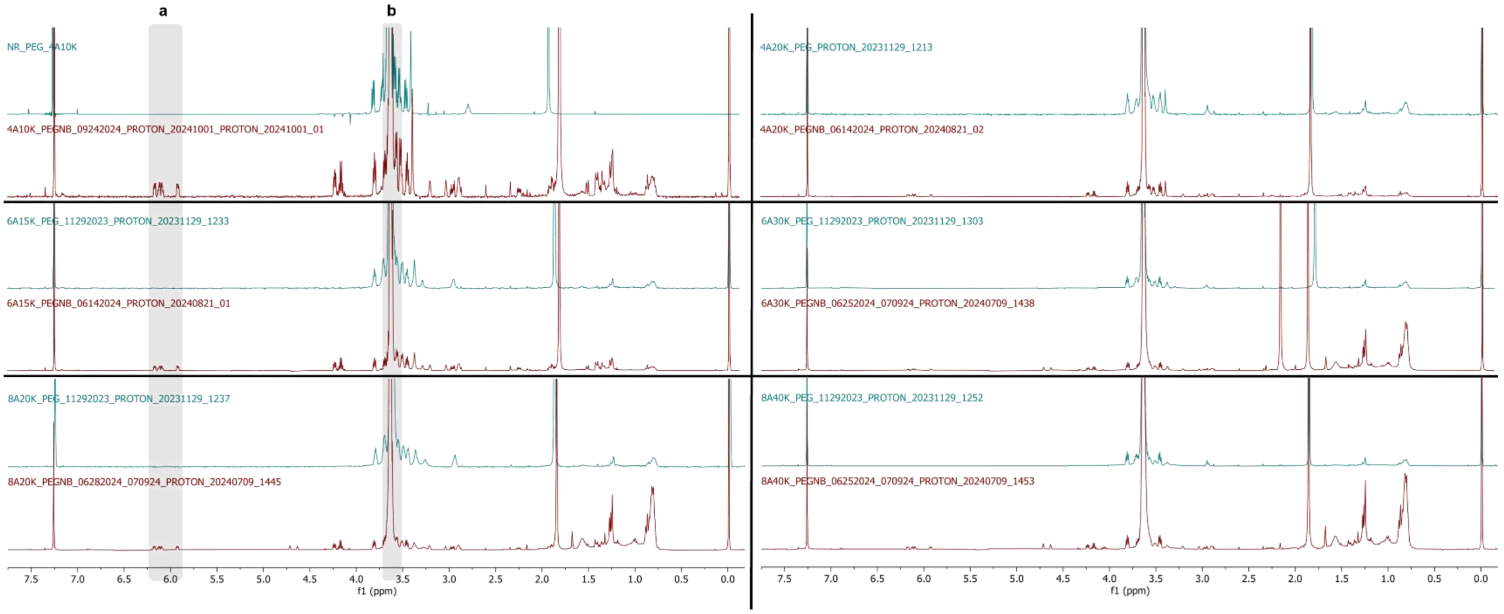

Supplementary Figure 2. H-NMR Spectra of PEG and PEGNB polymer formulations. a) 5.8-6.25 ppm (norbornene 4H, -C_7H_5) b) δ- 3.5-3.75 ppm (PEG backbone 1H, -CH-CH-O).

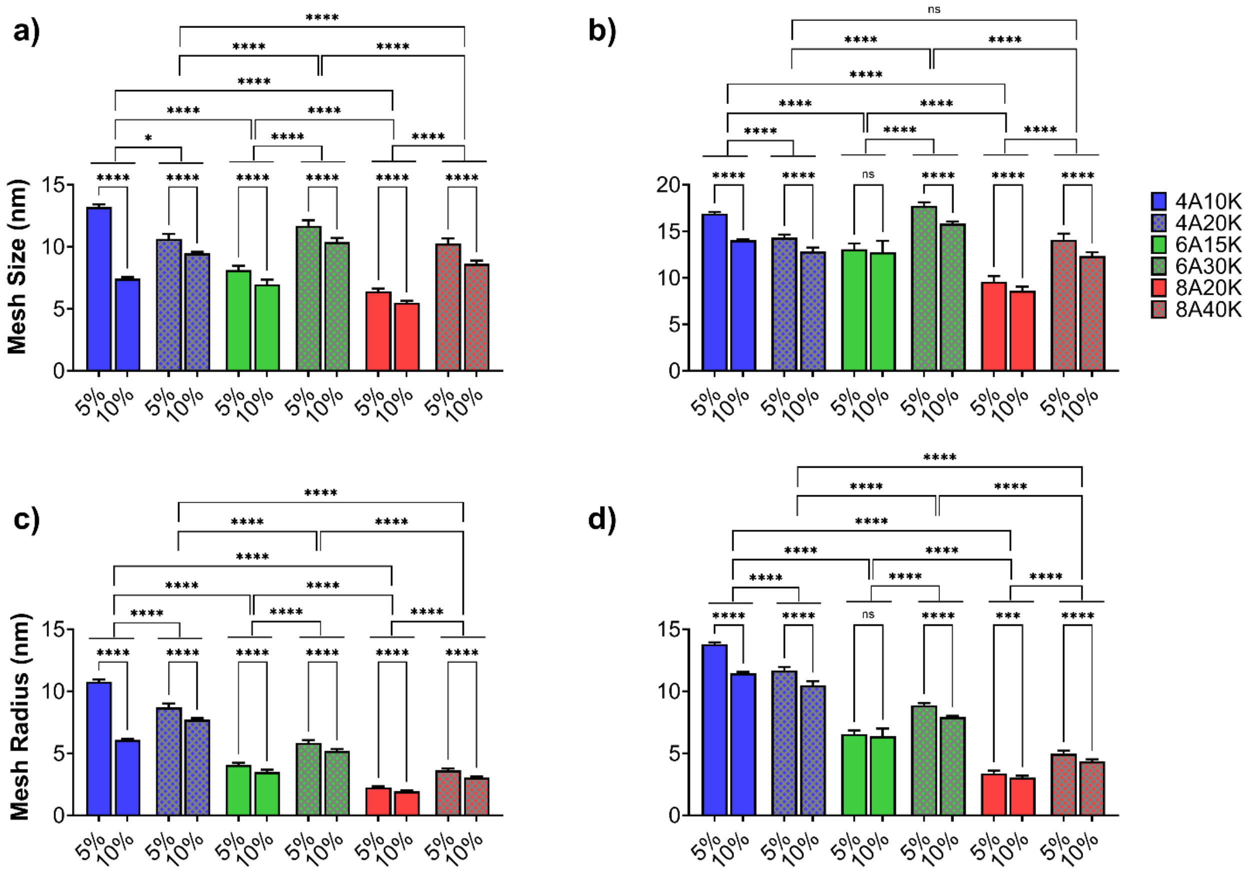

Supplementary Figure 3. (a,b) Mesh size and (c,d) mesh radius for PEGNB hydrogels crosslinked with (a,c) non-Degradable and (b,d) degradable crosslinker. In mesh size, the 6-arm formulation has the largest mesh size, followed by 8-arm, then 4-arm. In the mesh radius, 4-arm formulations generally have higher mesh radius, and the mesh radius decreases with the increase in number of arms. The derivation of mesh radius from mesh size illustrates the impact of network geometry on the hydrogel system that is overlooked in the original mesh size equation. (n=10-15).

Supplementary Table 1. The functionalization of PEG with norbornene groups calculated from the H-NMR spectra in SF1.

PEG Formulation	% Functionalization
4-arm 10 kDa	65%
4-arm 20 kDa	63%
6-arm 15 kDa	61%
6-arm 30 kDa	61%
8-arm 20 kDa	65%
8-arm 40 kDa	59%

Supplementary Table 2. Swelling ratio values.

Percent Polymer Volume	PEG Formulation	Non-Degradable Crosslinker		Degradable Crosslinker	
		Swelling Ratio (Mean ± SD)	n	Swelling Ratio (Mean ± SD)	n
5%	4-arm 10 kDa	1.45 ± 0.02	14	2.71 ± 0.14	15
	4-arm 20 kDa	1.78 ± 0.03	15	3.66 ± 0.09	15
	6-arm 15 kDa	1.06 ± 0.02	15	2.05 ± 0.11	15
	6-arm 30 kDa	1.54 ± 0.06	15	2.81 ± 0.22	15
	8-arm 20 kDa	0.89 ± 0.01	10	1.49 ± 0.06	15
	8-arm 40 kDa	1.36 ± 0.03	14	1.89 ± 0.08	14
10%	4-arm 10 kDa	1.85 ± 0.02	14	3.72 ± 0.06	15
	4-arm 20 kDa	2.48 ± 0.03	15	4.86 ± 0.10	15
	6-arm 15 kDa	1.52 ± 0.07	15	3.18 ± 0.08	14
	6-arm 30 kDa	2.11 ± 0.07	15	3.97 ± 0.07	15
	8-arm 20 kDa	1.27 ± 0.03	15	2.23 ± 0.07	14
	8-arm 40 kDa	1.56 ± 0.06	15	2.56 ± 0.09	15

Supplementary Table 3. Swelling ratio fold-change for PEGNB hydrogels made with non-degradable crosslinker.

	5% 4A10K	10% 4A10K	5% 4A20K	10% 4A20K	5% 6A15K	10% 6A15K	5% 6A30K	10% 6A30K	5% 8A20K	10% 8A20K	5% 8A40K	10% 8A40K
5% 4A10K		0.274	0.227	0.704	-0.271	0.042	0.060	0.453	-0.390	-0.129	-0.066	0.073
10% 4A10K	0.274		-0.037	0.338	-0.427	-0.182	-0.168	0.141	-0.521	-0.316	-0.267	-0.157
5% 4A20K	0.227	-0.037		0.389	-0.405	-0.150	-0.136	0.185	-0.502	-0.290	-0.239	-0.125
10% 4A20K	0.704	0.338	0.389		-0.572	-0.388	-0.378	-0.147	-0.642	-0.489	-0.452	-0.370
5% 6A15K	-0.271	-0.427	-0.405	-0.572		0.429	0.453	0.992	-0.163	0.194	0.280	0.472
10% 6A15K	0.042	-0.182	-0.150	-0.388	0.429		0.017	0.394	-0.414	-0.164	-0.104	0.030
5% 6A30K	0.060	-0.168	-0.136	-0.378	0.453	0.017		0.371	-0.424	-0.178	-0.119	0.013
10% 6A30K	0.453	0.141	0.185	-0.147	0.992	0.394	0.371		-0.580	-0.401	-0.358	-0.261
5% 8A20K	-0.390	-0.521	-0.502	-0.642	-0.163	-0.414	-0.424	-0.580		0.427	0.529	0.758
10% 8A20K	-0.129	-0.316	-0.290	-0.489	0.194	-0.164	-0.178	-0.401	0.427		0.072	0.232
5% 8A40K	-0.066	-0.267	-0.239	-0.452	0.280	-0.104	-0.119	-0.358	0.529	0.072		0.150
10% 8A40K	0.073	-0.157	-0.125	-0.370	0.472	0.030	0.013	-0.261	0.758	0.232	0.150	

Supplementary Table 4. Swelling ratio fold-change for PEGNB hydrogels made with degradable crosslinker.

	5% 4A10K	10% 4A10K	5% 4A20K	10% 4A20K	5% 6A15K	10% 6A15K	5% 6A30K	10% 6A30K	5% 8A20K	10% 8A20K	5% 8A40K	10% 8A40K
5% 4A10K		0.371	0.348	0.792	-0.244	0.173	0.035	0.464	-0.452	-0.177	-0.302	-0.058
10% 4A10K	0.371		-0.017	0.307	-0.448	-0.145	-0.245	0.068	-0.601	-0.400	-0.491	-0.313
5% 4A20K	0.348	-0.017		0.329	-0.439	-0.130	-0.232	0.086	-0.594	-0.390	-0.483	-0.301
10% 4A20K	0.792	0.307	0.329		-0.578	-0.346	-0.423	-0.183	-0.694	-0.541	-0.611	-0.474
5% 6A15K	-0.244	-0.448	-0.439	-0.578		0.550	0.368	0.935	-0.276	0.087	-0.078	0.245
10% 6A15K	0.173	-0.145	-0.130	-0.346	0.550		-0.118	0.248	-0.533	-0.299	-0.405	-0.197
5% 6A30K	0.035	-0.245	-0.232	-0.423	0.368	-0.118		0.415	-0.471	-0.205	-0.326	-0.090
10% 6A30K	0.464	0.068	0.086	-0.183	0.935	0.248	0.415		-0.626	-0.438	-0.523	-0.357
5% 8A20K	-0.452	-0.601	-0.594	-0.694	-0.276	-0.533	-0.471	-0.626		0.502	0.274	0.720
10% 8A20K	-0.177	-0.400	-0.390	-0.541	0.087	-0.299	-0.205	-0.438	0.502		-0.152	0.145
5% 8A40K	-0.302	-0.491	-0.483	-0.611	-0.078	-0.405	-0.326	-0.523	0.274	-0.152		0.350
10% 8A40K	-0.058	-0.313	-0.301	-0.474	0.245	-0.197	-0.090	-0.357	0.720	0.145	0.350	

Supplementary Table 5. Storage Modulus values obtained via rheology.

Percent Polymer Volume	PEG Formulation	Non-Degradable Crosslinker Storage Modulus (kPa) (Mean ± SD)	n	Degradable Crosslinker Storage Modulus (kPa) (Mean ± SD)	n
5%	4-arm 10 kDa	7.05 ± 0.15	14	4.64 ± 0.45	15
	4-arm 20 kDa	6.36 ± 0.42	15	3.55 ± 0.28	15
	6-arm 15 kDa	11.20 ± 0.34	15	11.19 ± 0.45	15
	6-arm 30 kDa	7.86 ± 0.50	15	5.49 ± 0.49	15
	8-arm 20 kDa	9.51 ± 0.32	15	12.30 ± 0.67	15
	8-arm 40 kDa	9.13 ± 1.40	15	7.89 ± 0.53	15
10%	4-arm 10 kDa	24.65 ± 1.86	15	15.66 ± 0.53	15
	4-arm 20 kDa	15.17 ± 1.24	15	8.94 ± 0.48	15
	6-arm 15 kDa	31.61 ± 2.66	15	26.21 ± 1.35	15
	6-arm 30 kDa	22.75 ± 1.48	15	14.74 ± 0.67	15
	8-arm 20 kDa	44.83 ± 1.51	15	32.71 ± 1.84	15
	8-arm 40 kDa	31.25 ± 2.41	15	20.76 ± 1.06	15

Supplementary Table 6. Rheology fold-change for hydrogels made with non-degradable crosslinker.

	5% 4A10K	10% 4A10K	5% 4A20K	10% 4A20K	5% 6A15K	10% 6A15K	5% 6A30K	10% 6A30K	5% 8A20K	10% 8A20K	5% 8A40K	10% 8A40K
5% 4A10K		2.494	-0.099	1.150	0.588	3.481	0.114	2.225	0.348	5.355	0.294	3.430
10% 4A10K	2.494		-0.742	-0.385	-0.546	0.282	-0.681	-0.077	-0.614	0.819	-0.630	0.268
5% 4A20K	-0.099	-0.742		1.386	0.762	3.972	0.237	2.579	0.496	6.052	0.436	3.915
10% 4A20K	1.150	-0.385	1.386		-0.262	1.084	-0.482	0.500	-0.373	1.955	-0.398	1.060
5% 6A15K	0.588	-0.546	0.762	-0.262		1.822	-0.298	1.031	-0.151	3.002	-0.185	1.790
10% 6A15K	3.481	0.282	3.972	1.084	1.822		-0.751	-0.280	-0.699	0.418	-0.711	-0.011
5% 6A30K	0.114	-0.681	0.237	-0.482	-0.298	-0.751		1.894	0.210	4.703	0.161	2.975
10% 6A30K	2.225	-0.077	2.579	0.500	1.031	-0.280	1.894		-0.582	0.970	-0.599	0.373
5% 8A20K	0.348	-0.614	0.496	-0.373	-0.151	-0.699	0.210	-0.582		3.715	-0.040	2.286
10% 8A20K	5.355	0.819	6.052	1.955	3.002	0.418	4.703	0.970	3.715		-0.796	-0.303
5% 8A40K	0.294	-0.630	0.436	-0.398	-0.185	-0.711	0.161	-0.599	-0.040	-0.796		2.423
10% 8A40K	3.430	0.268	3.915	1.060	1.790	-0.011	2.975	0.373	2.286	-0.303	2.423	

Supplementary Table 7. Rheology fold-change for PEGNB hydrogels made with degradable crosslinker.

	5% 4A10K	10% 4A10K	5% 4A20K	10% 4A20K	5% 6A15K	10% 6A15K	5% 6A30K	10% 6A30K	5% 8A20K	10% 8A20K	5% 8A40K	10% 8A40K
5% 4A10K		2.372	-0.235	0.926	1.411	4.646	0.181	2.176	1.648	6.046	0.700	3.472
10% 4A10K	2.372		-0.773	-0.429	-0.285	0.674	-0.650	-0.058	-0.215	1.089	-0.496	0.326
5% 4A20K	-0.235	-0.773		1.518	2.152	6.381	0.544	3.151	2.462	8.211	1.222	4.846
10% 4A20K	0.926	-0.429	1.518		0.252	1.931	-0.387	0.649	0.375	2.658	-0.118	1.322
5% 6A15K	1.411	-0.285	2.152	0.252		1.342	-0.510	0.317	0.099	1.923	-0.295	0.855
10% 6A15K	4.646	0.674	6.381	1.931	1.342		-0.791	-0.438	-0.531	0.248	-0.699	-0.208
5% 6A30K	0.181	-0.650	0.544	-0.387	-0.510	-0.791		1.688	1.242	4.964	0.438	2.785
10% 6A30K	2.176	-0.058	3.151	0.649	0.317	-0.438	1.688		-0.166	1.219	-0.465	0.408
5% 8A20K	1.648	-0.215	2.462	0.375	0.099	-0.531	1.242	-0.166		1.661	-0.358	0.689
10% 8A20K	6.046	1.089	8.211	2.658	1.923	0.248	4.964	1.219	1.661		-0.759	-0.365
5% 8A40K	0.700	-0.496	1.222	-0.118	-0.295	-0.699	0.438	-0.465	-0.358	-0.759		1.631
10% 8A40K	3.472	0.326	4.846	1.322	0.855	-0.208	2.785	0.408	0.689	-0.365	1.631	

Supplementary Table 8. Diffusivity coefficient values obtained from FRAP using fluorescein (300 Da) probe.

		Non-Degradable Crosslinker		Degradable Crosslinker	
Percent Polymer Volume	PEG Formulation	Diffusion Coefficient (cm^2/s) (Mean ± SD)	n	Diffusion Coefficient (cm^2/s) (Mean ± SD)	n
5%	4-arm 10 kDa	1.86E-06 ± 1.30E-07	27	1.27E-06 ± 2.08E-08	27
	4-arm 20 kDa	1.54E-06 ± 9.53E-08	27	1.30E-06 ± 4.42E-08	27
	6-arm 15 kDa	1.74E-06 ± 1.13E-07	27	1.08E-06 ± 6.26E-08	27
	6-arm 30 kDa	1.50E-06 ± 5.78E-08	27	1.29E-06 ± 3.82E-08	27
	8-arm 20 kDa	1.62E-06 ± 6.89E-08	19	9.71E-07 ± 2.90E-08	24
	8-arm 40 kDa	1.56E-06 ± 4.85E-08	27	1.22E-06 ± 2.67E-08	27
10%	4-arm 10 kDa	1.33E-06 ± 1.78E-08	27	1.22E-06 ± 4.26E-08	27
	4-arm 20 kDa	1.40E-06 ± 2.96E-08	24	1.28E-06 ± 6.09E-08	27
	6-arm 15 kDa	1.42E-06 ± 4.53E-08	27	1.08E-06 ± 3.78E-08	27
	6-arm 30 kDa	1.35E-06 ± 2.44E-08	27	1.21E-06 ± 3.70E-08	27
	8-arm 20 kDa	1.10E-06 ± 3.00E-08	27	9.66E-07 ± 2.45E-08	26
	8-arm 40 kDa	1.50E-06 ± 2.57E-08	27	1.19E-06 ± 4.48E-08	27

Supplementary Table 9. Diffusion Coefficient fold-change for hydrogels made with non-degradable crosslinker using fluorescein (300Da) probe.

	5% 4A10K	10% 4A10K	5% 4A20K	10% 4A20K	5% 6A15K	10% 6A15K	5% 6A30K	10% 6A30K	5% 8A20K	10% 8A20K	5% 8A40K	10% 8A40K
5% 4A10K		-0.283	-0.170	-0.249	-0.065	-0.237	-0.193	-0.277	-0.132	-0.407	-0.164	-0.197
10% 4A10K	-0.283		0.158	0.048	0.304	0.065	0.126	0.009	0.211	-0.172	0.166	0.121
5% 4A20K	-0.170	0.158		-0.095	0.126	-0.080	-0.027	-0.128	0.046	-0.285	0.007	-0.032
10% 4A20K	-0.249	0.048	-0.095		0.244	0.017	0.075	-0.037	0.156	-0.210	0.113	0.070
5% 6A15K	-0.065	0.304	0.126	0.244		-0.183	-0.136	-0.226	-0.071	-0.365	-0.106	-0.140
10% 6A15K	-0.237	0.065	-0.080	0.017	-0.183		0.057	-0.053	0.137	-0.223	0.094	0.052
5% 6A30K	-0.193	0.126	-0.027	0.075	-0.136	0.057		-0.104	0.075	-0.265	0.035	-0.005
10% 6A30K	-0.277	0.009	-0.128	-0.037	-0.226	-0.053	-0.104		0.200	-0.180	0.155	0.111
5% 8A20K	-0.132	0.211	0.046	0.156	-0.071	0.137	0.075	0.200		-0.317	-0.037	-0.074
10% 8A20K	-0.407	-0.172	-0.285	-0.210	-0.365	-0.223	-0.265	-0.180	-0.317		0.409	0.354
5% 8A40K	-0.164	0.166	0.007	0.113	-0.106	0.094	0.035	0.155	-0.037	0.409		-0.039
10% 8A40K	-0.197	0.121	-0.032	0.070	-0.140	0.052	-0.005	0.111	-0.074	0.354	-0.039	

Supplementary Table 10. Diffusion Coefficient fold-change for hydrogels made with degradable crosslinker using fluorescein (300Da) probe.

	5% 4A10K	10% 4A10K	5% 4A20K	10% 4A20K	5% 6A15K	10% 6A15K	5% 6A30K	10% 6A30K	5% 8A20K	10% 8A20K	5% 8A40K	10% 8A40K
5% 4A10K		-0.040	0.020	0.003	-0.150	-0.153	0.015	-0.052	-0.238	-0.242	-0.045	-0.068
10% 4A10K	-0.040		0.062	0.045	-0.115	-0.117	0.057	-0.012	-0.206	-0.210	-0.005	-0.029
5% 4A20K	0.020	0.062		-0.016	-0.167	-0.169	-0.005	-0.070	-0.252	-0.257	-0.063	-0.086
10% 4A20K	0.003	0.045	-0.016		-0.153	-0.156	0.012	-0.055	-0.240	-0.244	-0.048	-0.071
5% 6A15K	-0.150	-0.115	-0.167	-0.153		-0.003	0.194	0.116	-0.103	-0.108	0.124	0.096
10% 6A15K	-0.153	-0.117	-0.169	-0.156	-0.003		0.198	0.120	-0.100	-0.105	0.128	0.100
5% 6A30K	0.015	0.057	-0.005	0.012	0.194	0.198		-0.065	-0.249	-0.253	-0.059	-0.082
10% 6A30K	-0.052	-0.012	-0.070	-0.055	0.116	0.120	-0.065		-0.196	-0.201	0.007	-0.018
5% 8A20K	-0.238	-0.206	-0.252	-0.240	-0.103	-0.100	-0.249	-0.196		-0.006	0.253	0.222
10% 8A20K	-0.242	-0.210	-0.257	-0.244	-0.108	-0.105	-0.253	-0.201	-0.006		0.260	0.229
5% 8A40K	-0.045	-0.005	-0.063	-0.048	0.124	0.128	-0.059	0.007	0.253	0.260		-0.025
10% 8A40K	-0.068	-0.029	-0.086	-0.071	0.096	0.100	-0.082	-0.018	0.222	0.229	-0.025	

Supplementary Table 11. Diffusivity coefficient values for obtained from FRAP using FITC-Dextran 4 kDa probe.

Percent Polymer Volume	PEG Formulation	Non-Degradable Crosslinker		Degradable Crosslinker	
		Diffusion Coefficient (cm^2/s) (Mean ± SD)	n	Diffusion Coefficient (cm^2/s) (Mean ± SD)	n
5%	4-arm 10 kDa	9.03E-07 ± 1.04E-08	26	7.05E-07 ± 1.16E-08	27
	4-arm 20 kDa	7.83E-07 ± 8.05E-09	26	7.93E-07 ± 2.36E-08	26
	6-arm 15 kDa	6.46E-07 ± 1.84E-08	26	7.10E-07 ± 2.61E-08	26
	6-arm 30 kDa	7.34E-07 ± 1.15E-08	27	7.26E-07 ± 1.03E-08	27
	8-arm 20 kDa	6.83E-07 ± 3.29E-08	18	4.98E-07 ± 2.67E-08	27
	8-arm 40 kDa	8.38E-07 ± 3.00E-08	27	7.34E-07 ± 3.81E-08	27
10%	4-arm 10 kDa	6.28E-07 ± 9.92E-09	27	6.75E-07 ± 1.55E-08	26
	4-arm 20 kDa	7.47E-07 ± 1.44E-08	24	7.86E-07 ± 1.50E-08	27
	6-arm 15 kDa	6.72E-07 ± 9.92E-08	27	6.18E-07 ± 2.44E-08	27
	6-arm 30 kDa	6.71E-07 ± 8.91E-09	27	6.78E-07 ± 1.18E-08	26
	8-arm 20 kDa	5.65E-07 ± 2.09E-08	25	5.29E-07 ± 2.29E-08	27
	8-arm 40 kDa	7.69E-07 ± 1.41E-08	26	7.43E-07 ± 1.03E-08	27

Supplementary Table 12. Diffusion Coefficient fold-change for made with non-degradable crosslinker using FITC-Dextran 4 kDa probe.

	5% 4A10K	10% 4A10K	5% 4A20K	10% 4A20K	5% 6A15K	10% 6A15K	5% 6A30K	10% 6A30K	5% 8A20K	10% 8A20K	5% 8A40K	10% 8A40K
5% 4A10K		-0.304	-0.133	-0.173	-0.285	-0.256	-0.187	-0.257	-0.244	-0.374	-0.072	-0.148
10% 4A10K	-0.304		0.246	0.189	0.028	0.070	0.168	0.068	0.087	-0.100	0.334	0.225
5% 4A20K	-0.133	0.246		-0.046	-0.175	-0.142	-0.063	-0.143	-0.128	-0.278	0.071	-0.018
10% 4A20K	-0.173	0.189	-0.046		-0.135	-0.100	-0.017	-0.102	-0.085	-0.243	0.123	0.030
5% 6A15K	-0.285	0.028	-0.175	-0.135		0.041	0.136	0.039	0.058	-0.125	0.298	0.191
10% 6A15K	-0.256	0.070	-0.142	-0.100	0.041		0.092	-0.002	0.016	-0.159	0.248	0.145
5% 6A30K	-0.187	0.168	-0.063	-0.017	0.136	0.092		-0.086	-0.069	-0.230	0.142	0.048
10% 6A30K	-0.257	0.068	-0.143	-0.102	0.039	-0.002	-0.086		0.018	-0.157	0.250	0.147
5% 8A20K	-0.244	0.087	-0.128	-0.085	0.058	0.016	-0.069	0.018		-0.173	0.227	0.126
10% 8A20K	-0.374	-0.100	-0.278	-0.243	-0.125	-0.159	-0.230	-0.157	-0.173		0.483	0.361
5% 8A40K	-0.072	0.334	0.071	0.123	0.298	0.248	0.142	0.250	0.227	0.483		-0.082
10% 8A40K	-0.148	0.225	-0.018	0.030	0.191	0.145	0.048	0.147	0.126	0.361	-0.082	

Supplementary Table 13. Diffusion Coefficient fold-change for made with degradable crosslinker using FITC-Dextran 4 kDa probe.

	5% 4A10K	10% 4A10K	5% 4A20K	10% 4A20K	5% 6A15K	10% 6A15K	5% 6A30K	10% 6A30K	5% 8A20K	10% 8A20K	5% 8A40K	10% 8A40K
5% 4A10K		-0.043	0.124	0.114	0.006	-0.124	0.029	-0.039	-0.294	-0.250	0.041	0.053
10% 4A10K	-0.043		0.175	0.165	0.052	-0.084	0.076	0.004	-0.261	-0.216	0.088	0.101
5% 4A20K	0.124	0.175		-0.009	-0.105	-0.221	-0.084	-0.145	-0.371	-0.333	-0.074	-0.063
10% 4A20K	0.114	0.165	-0.009		-0.097	-0.214	-0.076	-0.138	-0.366	-0.327	-0.066	-0.055
5% 6A15K	0.006	0.052	-0.105	-0.097		-0.129	0.023	-0.045	-0.298	-0.255	0.035	0.047
10% 6A15K	-0.124	-0.084	-0.221	-0.214	-0.129		0.175	0.097	-0.194	-0.144	0.188	0.202
5% 6A30K	0.029	0.076	-0.084	-0.076	0.023	0.175		-0.067	-0.314	-0.272	0.011	0.023
10% 6A30K	-0.039	0.004	-0.145	-0.138	-0.045	0.097	-0.067		-0.265	-0.220	0.084	0.096
5% 8A20K	-0.294	-0.261	-0.371	-0.366	-0.298	-0.194	-0.314	-0.265		0.061	0.473	0.490
10% 8A20K	-0.250	-0.216	-0.333	-0.327	-0.255	-0.144	-0.272	-0.220	0.061		0.388	0.404
5% 8A40K	0.041	0.088	-0.074	-0.066	0.035	0.188	0.011	0.084	0.473	0.388		0.011
10% 8A40K	0.053	0.101	-0.063	-0.055	0.047	0.202	0.023	0.096	0.490	0.404	0.011	

References:

Richbourg, N.R., Ravikumar, A. and Peppas, N.A. (2021), Solute Transport Dependence on 3D Geometry of hydrogel Networks. Macromol. Chem. Phys., 222: 2100138. https://doi.org/10.1002/macp.202100138
